# Bilateral staged thoracotomy for multiple lung hydatidosis

**DOI:** 10.1186/1749-8090-8-121

**Published:** 2013-05-03

**Authors:** Leyla Hasdıraz, Omer Onal, Fahri Oguzkaya

**Affiliations:** 1 Department of Thoracic Surgery, Erciyes University Medical Faculty, Kayseri 38039, Turkey

**Keywords:** Hydatid disease, Lung, Thoracotomy, Multiple cysts

## Abstract

**Background:**

Hydatid cyst disease is still a problem in many countries. Surgical removal is currently the generally accepted choice of treatment for lung hydatidosis. However, operating on bilateral widespread lung hydatidosis is still controversial. The aim of this retrospective study was to evaluate the results of surgical treatment in bilateral multiple hydatid disease of the lung.

**Methods:**

In this study, we reviewed our experience in the surgical treatment of 17 (3.7%) patients with bilateral, and at least three, lung hydatid cysts. These 17 patients (8 male, 9 female), with an average age of 34.6 years (range 12–58 years), underwent bilateral staged thoracotomy.

**Results:**

In total 105 lung cysts were removed from 17 patients who underwent staged thoracotomies. The mean count of cysts was 6.7 (range 3–20 cysts). Most of the cysts (38.2%) were located in the right lower lobe. The mean interval between thoracotomies was 4.2 (range 3–5) days. Two patients (11.7%) had cysts associated with hepatic hydatidosis and one (5.8%) had cysts associated with the spleen; they were treated via phrenotomy during thoracotomies. All cysts were removed without lung resection. We observed some complications such as prolonged air leaks (n = 2), atelectasis (n = 3) and empyema (n = 2). No further surgery was required for management of complications. The mean hospital stay was 9.3 days. (range 7–23 days). Oral albendazole was started on the 2nd post operative day after the first thoracotomy in the dose of 10–20 mg/kg and was continued for 3 months with a gap of 1 week after each 21 days. No recurrences or deaths occured during the follow-up period.

**Conclusions:**

Although staged thoracotomy applied in 3–5 days after the initial thoracotomy increases the total hospital stay, it decreases the chance of possible complications can occur in cysts in the other lung when long intervals are preferred between the first and the second thoracotomy. In our experience, bilateral staged thoracotomy is an appropriate surgical option because morbidity rates are minimal and the hospital stay is acceptable for the treatment of bilateral widespread lung hydatidosis, even in patients who had a total of 20 hydatid cysts.

## Background

Hydatid disease is still a serious health problem in some Mediterranean countries, the Middle East, New Zealand, Australia, South Africa, and South America , where it is endemic [1,2]. Recently, the prevalence of the disease has increased in Europe and North America, due to increased immigration [3]. The lung is the second most commonly affected organ after the liver, with occurrences ranging from 10 to 40% [1]. Bilateral pulmonary hydatidosis accounts for 4% to 26.7% of all cases of pulmonary hydatidosis [3,4].

Surgical removal is currently the generally accepted choice of treatment for lung hydatidosis. Medical treatment is limited with patients who refuse surgical intervention or who are considered to be inoperable. Operating on bilateral widespread lung hydatidosis is still controversial. The aim of this retrospective study was to evaluate the results of surgical treatment in bilateral multiple hydatid disease of the lung.

## Methods

**Figure 1:**
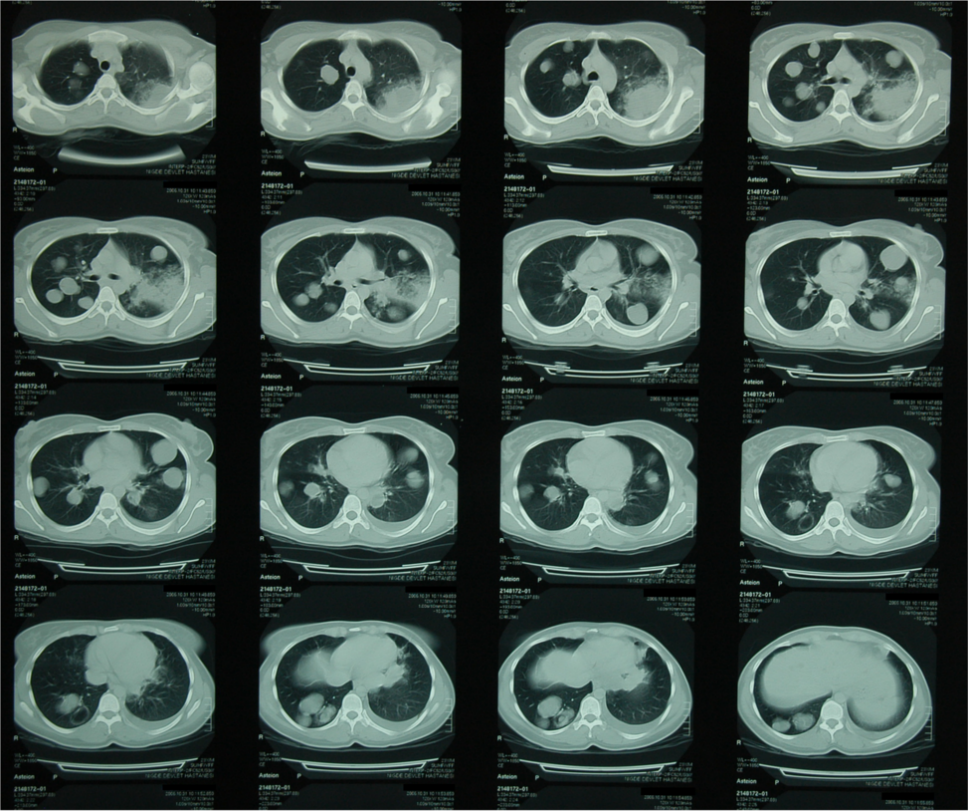
Computed tomography scan demonstrates bilateral multiple lung cysts.

Between January 1990 and January 2010, 452 patients with pulmonary echinococcosis were operated on at Erciyes University’s Thoracic Surgery Department. Of the 452 patients, 48 (10.6%) had bilateral lung hydatid cysts. We retrospectively reviewed the medical records of 17 (3.7%) patients who had bilateral, and at least three, lung cysts. None of these patients had intrathoracic but extrapulmonary located hydatid cysts such as diagrahmatic, pleural and mediastinal. 17 patients (8 male, 9 female) with an average age of 34.6 years (range, 12–58 years) underwent bilateral stage thoracotomy. All cases were studied thoroughly before surgery by means of clinical examination and investigations. Chest X-ray and computed tomography scan were the mainstays of diagnosis (Figure 1).

The study protocol was approved by institutional Ethics Committee (No:2013–150).

An abdominal ultrasound was performed in all cases as part of the preoperative evaluation. Baseline haematology, biochemistry, lung function tests and electrocardiogram were also carried out routinely in all cases.

Two stage bilateral thoracotomy was carried out under general anesthesia with double lumen endotracheal tubes. Muscle-sparing thoracotomies were carried out in all patients. A second muscle-sparing thoracotomy was carried out 3–5 days after the first thoracotomy. The side with the largest cyst or greatest number of cysts was initially treated. In patients who had an intact cyst on one side and a ruptured cyst on the other, the intact cyst was initially operated. All adhesions were totally divided to facilitate exploration and re-expansion of the lung. The operative field was isolated with 20% hypertonic saline pads for protection. Conservative parenchyma preserved surgical techniques were the methods of choice. While the lung was kept inflated, a 16-G needle connected to a suction tip was inserted into the cyst. After needle aspiration, an antiscolicidal agent (hypertonic saline 20%) was injected into the cystic cavity. We waited three minutes for deactivation of the cysts. When the cyst was aspirated and its fluid removed as completely as possible, the most prominent part of the cyst was opened (cystotomy) and the cyst membrane was removed with ring forceps. The cavity was then irrigated with saline solution and the bronchial openings were sutured. The cavity was obliterated with purse-string sutures of absorbable material, starting from the bottom (capitonnage) or its modifications. Anatomical resections were carried out in none of the patients. Patients with associated hepatic or splenic cysts were approached through the transthoracic transdiaphragmatic route.

Patients were extubated on the table and then shifted to the recovery room. The usual postoperative management was carried out including administering adequate analgesics and antibiotics. Inspiratory exercises were encouraged upon the first day following surgery. Chest drains were removed after cessation of drainage, air leak and radiological lung expansion.

Oral albendazole was started on the postoperative day two after the first thoracotomy in the dose of 10–20 mg/kg and was continued for 3 months with a gap of 1 week after each 21 days. Liver function parameters were monitored during the course of albendazole. The patients were followed up regularly. A chest X-ray was performed at the end of each year.

## Results

The most frequent symptoms were cough, chest pain and dyspnea (Table 1).A total of 105 lung cysts were removed from the 17 patients who underwent staged thoracotomies. Most of the cysts (36.1%) were located in the right lower lobe (Table 2).

**Table 1:** Symptoms of patients

**Symptoms**	**No. of patients (%)**
Cough	8 (47%)
Chest pain	6 (35%)
Dyspnea	5 (29.4%)
Blood-streaked sputum	3 (17.6%)
Haemoptysis	1 (5.8%)
Expectoration of salty sputum hydatid fluid and fragments of laminated membrane	**2 (11.7%)**
Hypersensivity reaction symptoms	† 2 (11.7%)
Febrile episodes	2 (11.7%)
Mucopurulent sputum	3 (17.6%)
Abdominal pain	2 (11.7%)

†Urticeria, pruritis and bronchospasm.

**Table 2:** Number of cysts according to location

**Patient no:**	**RUL**	**RML**	**RLL**	**LUL**	**LLL**	**Liver**	**Spleen**	**Total**
1	-	1	-	1	1	1	-	4
2	1	-	1	-	1	-	-	3
3	5	1	6	4	4	1	-	21
4	1	-	1	-	1	-	-	3
5	-	-	1	2	1	-	-	4
6	2	-	5	4	3	-	-	14
7	2	-	4	3	4	-	1	14
8	2	-	3	-	1	-	-	6
9	-	-	1	1	1	-	-	3
10	1	-	1	-	1	-	-	3
11	2	-	3	-	2	-	-	7
12	1	-	2	-	1	-	-	4
13	-	-	2	1	2	-	-	5
14	1	-	2	-	1	-	-	4
15	1	-	2	-	1	-	-	4
16	1	-	2	1	1	-	-	5
17	1	-	2	-	1	-	-	4
Total	21	2	38	17	27	2	1	108

*RUL*: Right upper lobe, *RML*: Right middle lobe, *RLL*: Right lower lobe, *LUL*: Left upper lobe, *LLL*: Left lower lobe.

**Figure 2:**
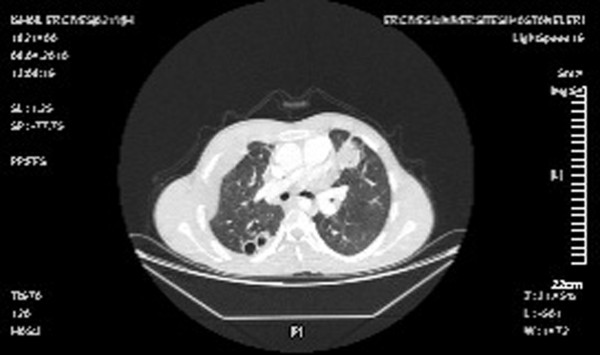
Computed tomographic scan of a 20-year-old male demonstrates bilateral multiple and perforated lung cysts four months after medical treatment.

The mean interval between thoracotomies was 4.2 (range 3–5) days. The mean cyst size was 3.8 cm (range 1–10 cm) in diameter. In 3 patients (17.6%), the cysts were suppurative. 37 of the cysts (35.2%) were perforated (Figure 2).

Cystotomy with capitonnage was carried out in all patients except in those with peripherally located small cysts. Additional surgical procedures such as wedge resection, decortication and debridement of necrotic tissues were carried out in 5 patients (29.4%). After removal of the cysts, the lung tissue was observed to have completely re-inflated in all patients. Postoperative complications such as prolonged air leaks, atelectasis and empyema were observed in 3 of 17 patients (Table 3). In two patients prolonged air leak and empyema occurred after the second thoracotomy. In empyema cases during surgery it was observed that the cysts were perforated and spilled into the pleural cavity. These patients with prolonged air leak and empyema received conservative medical treatment. In these two patients atelectasis occurred after the second thoracotomy and bronchoscopy was required. In one patient, atelectasis occured at early period of the first thoracotomy. It also recovered after bronchoscopy. Six cases in our series were referred to our clinic after failure of medical treatment. Three of them had a lung abscess. In one case, expansion of the lung could not be achieved so the first tube thoracostomy, which was applied in the first thoracotomy, was maintained during the second thoracotomy.

**Table 3:** Postoperative data of patients

**Variable**	**No. of patients (%)**
Prolonged air leak	2 (11.7%)
Empyema	2 (11.7%)
Atelectasis	3 (17.6%)
Wound infection	0 (0%)
Mean intensive care unit stay (days)*	1 day
Mean duration of tube thoracostomy (days)	4.7 (range 3–21 days)
Mean hospital stay (days)	9.3 (range 7–23 days)

*All patients were monitored in the intensive care unit in the first postoperative day.

No further surgery was required for management of complications. Associated abdominal hydatidosis was found in 3 (17.6%) cases. Two patients (11.7%) with cysts associated with hepatic hydatidosis and one (5.8%) with cysts associated with spleen were treated via phrenotomy during thoracotomy. All cysts were removed without lung resection. The mean hospital stay was 9.3 days. (range 7–23 days). Patients were restored to their full working capacity in a short period (2 ± 1 months). Neither recurrence nor death occurred during the follow-up period. (1–5 years).

## Discussion

The quests for minimally invasive treatment of bilateral widespread echinococcosis has continued for a long time. The potential risks involved in surgery may lead some physicians to prefer medical treatment. Albendazole is the most commonly used agent [5]. It is recommended that albendazole treatment should last for at least 3 months [3,6]. There are numerous side effects in medical treatment, which can be potentially life-threatening in some cases [7,8]. On the other hand, the number of cases reported where cysts disappeared as a result of this treatment is very rare [9]. While the clinical and radiological response to medical treatment is considered successful, about half of these cysts were found to be alive when patients underwent surgery [10]. On the other hand, although death of the cysts is seen as proof of the successful medical treatment, it is known that corrupt germinative membrane in the integrity of lung parenchyma can cause lung abscess.

Parenchymal sparing surgery is the generally preferred opinion in surgical treatment of echinococcosis. There is therefore no difference between removal of a large cyst or a large number of cysts within the same lobe as regards parenchymal loss after surgery. Essentially the idea that “the removal of a large number of cysts causes a capacity loss in lung parenchyma” is not proven.

In the literature, pulmonary function loss has not been shown in cases when a large number of cysts were removed from a lung. In our series, although we removed 6 cysts from the same lobe (a total of 20 cysts in both lungs) we observed that the lung ventilated very well at the end of the process. No radiological or clinical problems were seen in the postoperative period. On the other hand, parenchymal compression will disappear after removal of the cysts thus providing a higher lung volume than any loss resulting from surgical treatment. No technical reasons for inoperability, such as cysts location, were reported in patients who received surgical treatment for widespread lung hydatidosis. Therefore, the idea that aggressive surgical treatment for widespread hydatidosis is associated with morbidity is more likely associated with incision-related problems. Undoubtedly, the functional and aesthetic loss created by incisions can’t be ignored. However the effectiveness of medical treatment is very limited and it has potential side effects. Many diseases located in the thorax including hydatidosis can be afforded for radical surgical treatment to provide complete eradication of hydatidosis.

The surgical options for patients who have bilateral lung disease, include bilateral simultaneous thoracotomies (synchronous), bilateral staged thoracotomies separated by some time period (metachronous), median sternotomy and Clamshell incision. Each strategy has advantages and disadvantages and, like other controversial aspects of the care of these patients, has not been extensively studied. Probably the most common approach is staged thoracotomies, which provides optimal exposure of each hemithorax and avoids the longer operative and anesthetic time associated with synchronous thoracotomies. A disadvantage of this approach is the potential for delay between the first and second thoracotomies due to slow recovery or postoperative complications. This may allow progression of disease, making eventual resection more difficult [11]. Surgical treatment of pulmonary hydatid cysts may cause potantial complications especially in patients who have multiple bilateral lung cysts. Each surgical application has complication risks for bilateral lungs. The possibility of occurring complications in bilateral lung at the same time can be life threatening. So simultaneous bilateral lung complications may increase the mortality rates. Therefore we avoided to apply bilateral thoracotomies as a single stage.

Two stage thoracotomy and median sternotomy are the most common approaches in the surgical treatment of bilateral lung hydatidosis. Single-session surgery via median sternotomy is a rational intervention for bilateral lung hydatidosis. It provides high postoperative patient comfort and shorter duration of hospital stay [12,13]. However, the sine qua non rule in surgical treatment is to ensure the complete eradication of cysts. Therefore if some cysts can’t be reached for technical reasons via median sternotomy, surgical treatment can be unsuccessful. Prolonged air leak, postoperative lung and pleura infections are more likely to occur in cases of complicated cysts. Therefore, some clinics, including ours’, prefer two stage thoracotomy in cases in which cysts are difficult to reach and are complicated [13-15]. A few days after the first thoracotomy, the second stage thoracotomy can be performed after monitoring for surgical complications and reserves of the lungs to guarantee the health of the operated lung. We applied the second thoracotomies after anesthesiologist’s approval. Sevofluran was choosen for anesthesia because of its rare hepatotoxicity rate as mentioned in the literature [16].

Pulmonary hydatid cysts also have potentially life threatining complications such as infection and anaphylaxis.Therefore we endeavored not to loose time between two thoracotomies for patients’ safety. Already the complications occurred in two patients (prolonged air leak and empyema) were probably due to pleural perforation of pulmonary cysts. In these patients, intact side of cysts was preferred for the first thoracotomy.

On the other hand, the possibility of associated abdominal hydatidosis is very high in patients with bilateral diffuse cystic disease [4,17]. These diseases can be treated by adding phrenotomy to thoracotomy. In our series, we managed to succesfully treat two patients who presented with hepatic cysts and one patient with splenic cyst via phrenotomy. Even though we acknowledge that median sternotomy is a potentially effective approach, we did not use this approach in our series because it has been reported in the literature that mediastinal infections after median sternotomy may have mortal consequences [15].

In our cases we found it sufficient to wait 3–5 days between the two thoracotomies. We believe that this time is adequate to observe any complications which may occur after the first thoracotomy. The complication rate of our series was 17,6%. In our opinion this relatively high complication rate was due to pleural perforation and large size of cysts.

Surgical procedure-related mortality rates have been reported in the literature as 1–2% [4,18]. These mortality rates are acceptable, and the recurrence rate is low (1–3%).

In our series, we observed no mortality or recurrence in the follow up period. (1–5 years).

Limitation of this study is being a retrospective review including low number of patients. This study requires to be supported by large-scale prospective evaluations.

## Conclusions

Although two stage thoracotomy applied 3–5 days after the first thoracotomy increases the total hospital stay, it prevents possible complications that can occur in the other lung when long intervals are preferred before applying the second thoracotomy. In addition, it eliminates the necessity for re-assessment procedures before the second application because patients have not been discharged. In our experience, bilateral two stage thoracotomy is an appropriate surgical option with acceptable morbidity rates and duration of hospital stay for treatment of bilateral widespread lung hydatidosis, even in patients who had a total of 20 hydatid cysts.

## Consent

Written informed consent was obtained from the patient for publication of this report and any accompanying images.

## Abbreviations

RUL: Right upper lobe; RML: Right middle lobe; RLL: Right lower lobe; LUL: Left upper lobe; LLL: Left lower lobe.

## Competing interests

The authors declare that they have no competing interests.

## Authors’ contributions

All authors participate the surgical applications. LH participated in the design of the study and performed the statistical analysis. OO participated in extraction of the patients’ data and review of the literature, FO conceived of the study, and participated in its design and coordination and helped to draft the manuscript. All authors read and approved the final manuscript.
